# The TRAPD approach as a method for questionnaire translation

**DOI:** 10.3389/fpsyt.2023.1199989

**Published:** 2023-06-15

**Authors:** Peggy Walde, Birgit Angela Völlm

**Affiliations:** Clinic of Forensic Psychiatry, Rostock University Medical Center, Rostock, Germany

**Keywords:** questionnaire translation, TRAPD approach, forensic mental health, forensic psychiatry, restrictiveness

## Abstract

Surveys and questionnaires are widely used in various areas of psychological and psychiatric research and practice. Many instruments have been used in several languages and across cultural contexts. A popular method of choice for their translation into another language involves translation and back-translation. Unfortunately, this method’s ability to detect flaws in translation and necessities for cultural adaptation is limited. To address these shortcomings, the Translation, Review, Adjudication, Pretest, and Documentation (TRAPD) approach of questionnaire translation from cross-cultural survey design has been developed. In this approach, several translators with different professional backgrounds translate the questionnaire on their own first and then come together to discuss their versions. Since the translators’ expertise is required to vary (e.g., survey methodology experts, translation experts, expert knowledge in the questionnaire’s topic) the team approach results in a high-quality translation as well as offering opportunities for cultural adaptation. This article illustrates the application of the TRAPD approach on the basis of the translation process of the Forensic Restrictiveness Questionnaire from English into German. Differences and advantages are discussed.

## 1. Introduction

Questionnaires are one of the most frequently used instruments in psychological research and the social sciences in general. They are, for example, used to explore medical symptoms, personality characteristics, attitudes, feelings and other modalities that are difficult to assess otherwise. In most cases, questionnaires are originally developed to explore a phenomenon in a certain country or culture. Subsequent translations often require a number of adaptations to meet the characteristics of the new context (1).

The translation of a questionnaire is not just a translation of words. A questionnaire is intended to measure certain latent constructs that cannot be observed themselves. Therefore, the quality of the items determines if the construct is met and if the questionnaire is understood by the intended users. Both are crucial to generate valid results from which meaningful conclusions can be drawn. Mistakes in translation can add to misleading results and, in the worst case, wrong conclusions and decisions in research and practice. Therefore, linguistic characteristics (e.g., word order, sentence structure), cultural context (e.g., special connotations of single words, idioms) and knowledge about the population where the questionnaire is intended to be used (e.g., psychiatric patients, children, employees in a certain kind of company) have to be considered (2). Furthermore, the way the questionnaire is translated depends on its intended use. For some purposes, e.g., diagnostic, it is important that the questions fit well into the targeted culture and language. There are several diagnoses in the DSM-V that include a paragraph regarding culture-related diagnostic issues that need to be considered or, if not, might lead to inappropriate diagnoses. One example is the diagnosis of schizophrenia. The authors state that “Ideas that appear to be delusional in one culture (e.g., witchcraft) may be commonly held in another. In some cultures, visual or auditory hallucinations with a religious content, (e.g., hearing God’s voice) are a normal part of religious experience” [(3), p. 103].

Another often-cited example is the question of a person’s intelligence. What is or is not seen as part of intelligence varies across countries [e.g., (4)]. These examples clearly demonstrate, that questionnaire adaptations might be necessary, depending on the instrument’s purpose. If critical decisions are made based on the results of the questionnaire, more extensive adaptations can be useful and the translated instrument might differ from the source questionnaire to a larger extent (e.g., in psychiatric diagnostics or prognosis that informs decisions about release of offenders from detention). If the emphasis is more on cross-cultural comparability, it might be useful to limit adaptations to maintain the comparability of questions (5). In her publication Behr (6) points out five major challenges a professional translator is confronted with when translating a (psychological) questionnaire: Missing context in stand-alone items, missing knowledge about the construct measured, missing knowledge of intentions behind certain wording decisions, uncertainty about participants addressed (e.g., in adaptive testing, certain sets of items are limited to subgroups of participants, e.g., women or unemployed people), and uncertainties about leeway in standardizations (esp. when cross-cultural comparability between source and target language questionnaire is intended). All of these uncertainties can lead to invalid translations. With these thoughts in mind, one could ask if the translation through an experienced professional could be a better option. It is true that a person who is experienced with the questionnaire topic (e.g., a researcher, clinician, teacher etc.) and is fluent in the source language might be able to compensate the missing background knowledge of a professional translator. But compared with professional translators, these people will probably have issues with the linguistic nuances like lexis, connotations or sentence structure, that is not always obvious to (non-)native speakers without the corresponding linguistic background. Another field of expertise is the methodological knowledge about questionnaire design. Neither linguists nor specialists with language skills can be expected to have experience in questionnaire development and adaptation. Knowledge like that might, however, be relevant, e.g., in terms of translation of labels of Likert scales, comparable length and difficulty of items etc. In conclusion it would appear that it needs the expertise of at least three different professions to properly adapt a questionnaire. The need for different expertise is currently also mentioned by different questionnaire and survey guidelines [e.g., (7, 8)].

The current methods used in questionnaire translation include various forms of forward and backward translation. Forward translation describes the simple translation from a source language into a target language. A forward translation without any further measures of quality assurance is prone to errors and should be avoided (9). The most popular method to ensure translational quality of their questionnaire is backtranslation (10), i.e., the translation of the questionnaire back into its original (source) language. The original text and the backtranslation are compared. If differences in original and backtranslated questionnaires occur, this is seen as an indication for translation shortcomings. The process of backtranslation is also mentioned in the current version of the International Test Commission’s guidelines on translating and adapting tests (7). However, these guidelines also mention several shortcomings of forward and backtranslation when used alone. A combination of multiple translation approaches is recommended to overcome these. It is also mentioned that the translational process shall consider linguistic, psychological, and cultural differences between source and target questionnaire. However, the ITC guidelines do not describe specific translation procedures, so that the precise steps to be taken to address these processes remain unclear. As described, backtranslation is currently a popular method of quality assurance. At the same time this method has been critically reviewed by some experts. Behr (11) summarizes several shortcomings of backtranslation in her work. She mentions, e.g., that translational mistakes cannot be made during forward translation only but also during backtranslation. Furthermore, if backtranslation is used as part of a translation process, it might lead to translations that orient too closely on the source text. Difficulties can arise here in relation to necessary cultural adaptations which consequently lower the validity of the target questionnaire. Further issues can arise when forward and backward translators are not aware of the context or technical terms used in special fields of a questionnaire and therefore both use the wrong term. As example the author mentions a large cross-European survey in which the translators were asked to translate the English terms ‘child care service’ and “longterm care service” into German. The author described the issues arising during the process as follows:

“Care services” was translated into German as “Pflegedienste,” which was returned as “care services.” Thus, for back translation evaluators at least, all seemed fine. However, while in English one general term for both services is appropriate, in German you will have to be more careful. Depending on how you translate the ‘care’ bit, you may focus on caring for the ill and the elderly or you may focus more in general on attending to someone, regardless of ill health. The German term “Pflegedienst” for “care services” did not fit the questionnaire context since it is only used in the context of the ill and/or the elderly and is thus not fitting to general child care services” [(11), p. 580].

To overcome these shortcomings, several options exist. One alternative, also mentioned by the ITC, can be a forward translation by several independent translators and the reconciliation of their versions by a third, independent translator or expert (7). It is not stated in more detail, how this reconciliation should be done, e.g., if the third translator should meet with the other translators to discuss their versions or should do the translation by themselves only, if reconciliation should be done by a single person or a team and which qualification(s) these people should have. Acquadro et al. (12) describe various forms of synthesis of the several forward translations by the original translators, independent translators or (focus) groups with different people, some including, e.g., participants of the questionnaire’s intended user group. Interestingly, almost every translation process reviewed by Acquadro et al. included a backtranslation after the synthesis and an additional review of the results. The synthesis is regularly done by translators, whereas, e.g., methodologists, health care professionals or other experts are often involved in the review process. These procedures involve many different people and therefore make the translation process very time consuming and expensive.

**Figure 1:**
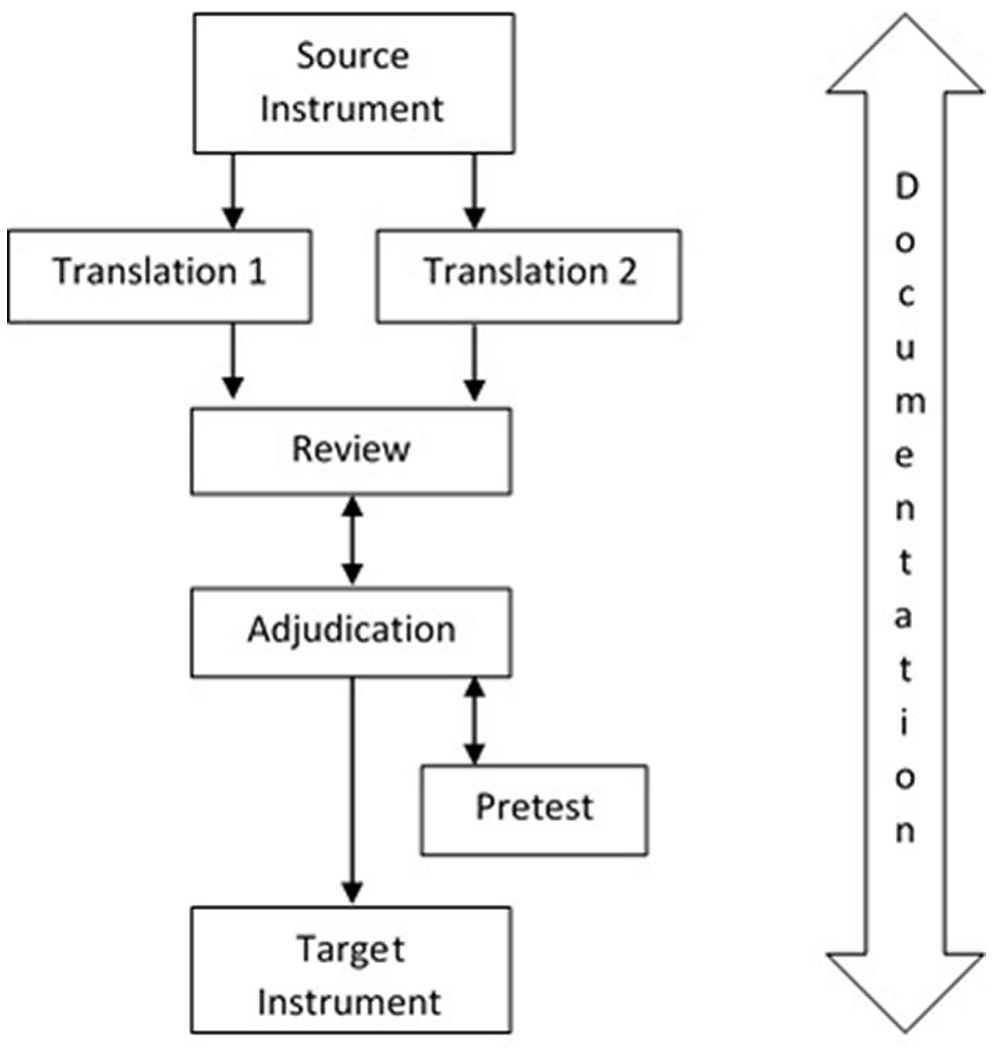
TRAPD team translation model [(13), p. 292].

Therefore, the TRAPD approach has been suggested by Harkness (13). Therein, TRAPD is an acronym for the steps of the translation process, specifically Translation, Review, Adjudication, Pretest, and Documentation (see Figure 1).

The TRAPD approach is meant to work with at least two translations from different translators. Ideally, each translator provides a draft translation of the whole questionnaire (parallel translation). If necessary, it is possible to let different translators translate different parts of an instrument, e.g., if the questionnaire is very extensive (split translation). In this case a certain amount of overlap is recommended (13). According to the author, the translations should be done by professional translators with experience in the topic of the questionnaire. If, e.g., a psychological questionnaire is translated, a translator with prior experience in education or legal translation might not be an appropriate choice. If it is difficult to find an appropriate translator, e.g., when language and/or topic are very specific, it is reasonable to draw on proficient bilinguals. The translators create a translation independent from each other and note issues or questions that might arise during the process. In the next step the several drafts are taken into a review discussion. All translators are part of an extended review team, along with experts for questionnaire methodology. Organizations using the team approach also involve participants in the expert review who are familiar with the questionnaire’s topic (9). Within a review meeting, one of the participants functions as senior reviewer who moderates the discussion and, as that, takes care of organizational duties (e.g., sticking to the schedule). During the review the various translation options of the drafts are compared with each other which can foster discussion. First adaptations can be made at that stage. In addition, mistakes of a single translation can be detected easily. Harkness (13) also states that contributions from several translators make it easier to deal with regional variances and individual preferences that might influence a translator’s decision. The opportunity to think about the translation in advance and being aware of its challenges also makes discussions more effective (p. 293). Furthermore, queries that might have occurred during translation are considered in the discussions. The result of the review process should be a single version of the translated questionnaire synthesized from the various drafts. If questions arise during the review process that cannot be resolved by the reviewers or if reviewers cannot agree on a translation, these are documented and postponed to the Adjudication phase. Depending on, e.g., the length of the questionnaire, one or more adjudicators can be involved. An adjudicator should be familiar with the translation project, methods and the questionnaire topic but was not involved in the actual translation process so far. The adjudicator(s), along with the review moderator, decide about the reviewed version and further adaptations and if the questionnaire translation is ready for pretesting. If the pretest reveals further adaptation necessities or other shortcomings, these are sent to the adjudicator in order to resolve them. There are several points of this approach that can be adapted to the individual circumstances of a project. Such adaptations are pointed out in the method section. The following text describes the application of the TRAPD approach to the translation of a questionnaire for forensic mental health research. We will describe in detail the procedure of each of the single steps (translation, review, adjudication, pretest, documentation) and point out individual adaptations of the process for our specific research context.

## 2. Method

### 2.1. Research design overview

The Forensic Restrictiveness Questionnaire [FRQ; (14)] has been developed and validated in the United Kingdom, UK. The FRQ is a questionnaire for forensic mental health in-patients to describe their subjective experience of restrictions that result from their detention in a forensic hospital. Tomlin (14) described the experience of restrictiveness as “The extent to which phenomena created, maintained or augmented directly or indirectly by forensic psychiatric care are subjectively experienced by a resident as infringing negatively upon their autonomy, self or personhood” (p. 253). Describing the experience of restrictiveness that way, the definition covers the experience of obvious restrictions (e.g., like locked doors, limited contact to people outside the hospital) as well as more subtle ones (e.g., limited privacy in double rooms, limited choice in daily routines and activities). The FRQ is a self-rating questionnaire with 10 questions regarding sociodemographic characteristics and 15 items (e.g., *The restrictions on the ward make sense*). Patients are asked to rate their agreement or disagreement for each of the 15 items on a 5-point Likert scale from 1 (strongly disagree) to 5 (strongly agree). The complete source questionnaire can be provided by the authors upon request. 

#### 2.1.1. Researcher description

The research team was composed of one female psychologist and PhD candidate student who worked as a researcher at the Clinic for Forensic Psychiatry at Rostock University Medical Center, Germany, and the female medical director of a Clinic of Forensic Psychiatry who was also holding a professorship in forensic psychiatry at Rostock University Medical Center. The medical director was one of the participants in the translation process and had been living and working Great Britain for almost two decades. The medical director’s history allowed an extensive understanding of the language and culture of both countries and knowledge about the forensic mental health system in both countries.

#### 2.1.2. Participants

The translation into German took place in 2020. We used the team approach introduced by Harkness (13). All of the four participating translators (3 females, 1 male) were born in Germany (one with a Turkish migrant background) and described German as their native language but lived or had been living and working/studying in the United Kigdom for between five and 19 years. Therefore, all of them were considered to have sufficient knowledge of the British and German culture additional to their language skills. None of the persons were professionally trained translators. Two of them were psychiatrists with several years of occupational experience in general and forensic psychiatry in the United Kigdom and Germany. The other translators had a degree in Psychology and in Pharmacy. Therefore, the translators combined expertise in British and German culture, (forensic) psychiatry in both countries and methodological knowledge regarding questionnaire design. The fourth translator with the pharmaceutical background was seen as lay translator. We expected the missing psychological-psychiatric background to add to a more everyday oriented translation of the questionnaire that is easier to understand for patients.

Participating patients were 17 forensic mental health patients treated in the Clinic of Forensic Psychiatry at Rostock University Medical Center. Nine of them participated in the first round, 8 in the second round. Five patients were detained according to § 63 Strafgesetzbuch (StGB, German penal code) which affects individuals who committed crimes because of a serious mental illness (e.g., schizophrenia or personality disorder). Detention under this paragraph is not limited in time but is evaluated annually to determine whether criteria for detention are still fulfilled. Another 12 patients were detained according to § 64 StGB, which refers to offenders with substance use disorders. Duration of detention under this paragraph is time limited, usually to 2 years and reviewed every 6 months. All but one patient were male. Patients’ age ranged from 20 to 62 years (*M* = 35.25, *Mdn* = 31, *SD* = 12.20), the typical age range for forensic mental health patients in Germany [Statistisches (15)]. Fourteen participants had a diagnosis of substance use disorder, seven of personality disorder, two of schizophrenia, one of intellectual disability and five of other diagnoses (comorbidities included). Their cognitive abilities ranged from 66 to 116 (*M* = 84.41, *Mdn* = 74, *SD* = 17.97) according to the German version of the Wechsler Adult Intelligence Scale [WAIS-IV, (16)] Two patients had a migration background. Both of them identified themselves as acculturated predominantly to German culture (see next paragraph for more details). A more detailed description of the sample is presented in Appendix A.

### 2.2. Participant sampling and recruitment

For the translational process, several attempts to recruit translators were made. We were looking for German native speakers who live or were living in Great Britain for at least 2 years and, therefore, were considered familiar to the British language and culture. Additionally, familiarity with (forensic) mental health care systems in both countries were desirable but not mandatory. In fact, it was turned out to be very difficult to find people with these characteristics. To recruit translators, members of the research team contacted people from their professional and personal background and also posted a call on various Facebook study participation groups. Four people were willing to participate. They were invided and offered a compensation for their time.

Patient participants for the cognitive pretest interview were patients of the Clinic for Forensic Psychiatry in Rostock, Germany, who were recruited in two rounds. The first round was the initial cognitive pretest conducted in late 2021 which already revealed adaptation necessities. The second round was conducted in early 2022. All participants were preselected by the research team according to a quota sampling plan. This plan contained several characteristics to make sure that also small but persistently existing subgroups are represented in the pretest that would have been underrepresented in random sampling (e.g., women). Characteristics that were taken into consideration for the quota plan were age, sex, diagnosis, legal basis for detention, and intellectual abilities WAIS-IV. We also included patients with migrant background (meaning the patient themselves or at least one of their parents were born and raised outside Germany). For these patients, a participation in a German questionnaire translation and validation process only made sense if the person showed a certain level of acculturation to German culture. Acculturation describes the individual process cultural change by becoming familiar and identifying oneself with the culture a person is living in [i.e., its behaviors, traditions, language etc., (17, 18)]. We used the Frankfurter Akkulturationsskala [Frankfurt Acculturation Scale; FRAKK, (18)] to identify patients who described themselves as acculturated to German culture. The FRAKK is based on a two-dimensional model of acculturation that states that a person can be acculturated in two cultures at the same time. Therefore, being involved in one’s culture of origin not necessarily excludes being involved in the majority society’s culture (or vice versa). The model finally states four different states – acculturation to origin culture or the majority society’s culture only, acculturation to both cultures or to none of them. For our cognitive pretest we tested patients with a migration background, who were interested in participating on the pretest with the FRAKK and invited patients who described themselves as acculturated to German culture alone or together with their (parents’) culture. We did not include patients who did not speak German to a level that enabled them to express themselves in an interview with open questions. We also excluded patients who were not able to give their informed consent for participation (e.g., because of active psychosis) and patients from local prisons who were in the clinic only for a short-term psychiatric intervention. Patients were offered 5€ for their participation.

### 2.3. Data collection

The translators were given information about the population the questionnaire was intended to address before the translation. This information contained a definition of forensic psychiatry, a brief overview of the forensic psychiatric system in Germany as well as the most important patient characteristics (e.g., average age, gender, education and diagnoses). All translators were asked to provide a translation that orients closely to the original questionnaire and its simplicity and avoid too complex translations due to the usually low level of educational achievement in the forensic population. The translators were provided a form to fill in their translation for every item of the FRQ and every sentence of the questionnaire instructions as well as space for alternative translations or notes about uncertainties that arose during translation. The four translators then prepared their translation independently from each other and sent them to the reviewer in advance of the review discussion. All translators translated the entire instructions and all items of the FRQ [parallel translation, (19)]. The reviewer, which was represented by the first author (PW) then prepared the translations for the review discussion.

The review discussion lasted 70 min. Unfortunately, the psychological translator was prevented at short notice and not able to join the review discussion, so four people attended (three translators and the reviewer as moderator). The discussion was held as a video conference given one of the translators was still living in the United Kingdom at that time. The reviewer prepared a presentation which presented one introductory sentence or item with its four translation versions on each slide on a shared screen. She welcomed the translators and introduced the aim of the discussion, which was to discuss the four different translations and, ideally, agree on a final version. She also pointed out the option to come up with a new version that evolved during discussion. The discussion was recorded and transcribed verbatim. Participants were able to agree on one translation version with the exception of two introductory sentences and one item. The decision regarding these parts was made by the reviewer in a discussion with the second author (BV), who served as adjudicator. Discussion was held 1 day after the review taking into account the reviewers’ notes and the audio record. Additionally, two professional translators were shown the original questionnaire as well as the review translation and asked for their evaluation. Their annotations were discussed by the reviewer and the adjudicator and final adaptations were made.

The review discussion as well as the evaluation of the translators left some questions regarding ambiguous wordings in the source questionnaire and the original author’s intention. These were solved by the reviewer via consultation of the original author or were taken into account as questions for the pretest which involved the intended user group, i.e., forensic patients. The preliminary version for the pretest was produced by the reviewer together with an adjudicator and checked for grammatical and spelling mistakes.

The version produced by the reviewer and adjudicator was taken to a cognitive pretest with forensic mental health in-patients. The pretest was designed as a semi-structured face to face interview. All interviews were conducted by the first author. The cognitive pretest consisted of an interview manual and prepared score cards. Patients were presented the introductory section of the FRQ and all of the 15 items one by one on a score card which was read out by the interviewer at the same time. After each score card, the interviewer first waited for patient’s initial reaction (e.g., questions or mimic that might indicate issues of comprehension). For the items, patients were then asked for their rating of each item. An additional score card with the 5-point Likert scale was on display all the time to support the answering process. For some of the items we used additional questions, so-called probes, to further explore patients’ answers and how they came to their conclusion. We used comprehensive probes, category selection probes and specific probes. Comprehensive probes are used to explore patients’ understanding of a certain word or phrase (e.g., *Could you please explain what* Individuum *means for you*?). Category selection probes were used to see how patients came to their answer (e.g., *So you chose a* 4 *in your last rating. Could you please explain why you chose this rating?*). Specific probes are used to explore more specific questions (e.g., *What hobbies were you thinking of, when you answered the question?*). If probes were not sufficient to elucidate the question, the interviewer was free to ask additional questions. Patients were informed at the beginning of the interview, that they can decline answering certain questions without giving a reason. The interviews were intended to last between 30 and 45 min. Their actual duration varied between 16 and 64 min. After the interview, the interviewer thanked the patients and handed over the expense allowance.

### 2.4. Data recording, transforming, and analysis

The review discussion was held as online conference via Zoom and was recorded via the conference’s recording function. The audio file was transcribed verbatim by a student assistant and checked for correctness by the first author.

For the cognitive pretest, the interviews took place in the visiting room of the clinic or in patients’ rooms, according to the patients’ preferences. All interviews were recorded on an audiotape and partially transcribed. We left out transcription of parts that had no relation to the pretest questions. All transcripts were analyzed using NVivo 12 pro according to predefined questions using Thematic Analysis (20).

## 3. Results

### 3.1. Translation process

Several challenges arose during the translation process. In some cases, the team had to weigh up a translation as close as possible to the original text against an adapted version. The first option would lead to better compatibility between the British and German version in international comparability studies. The latter option might lead to more valid data in a German population. If in doubt, the priority was given to validity in the German population and, therefore, to a good fitting adaptation, even if this might limit the international comparability of the British and German questionnaire versions.

The instruction sentences lead to several divergent translations. The first sentence of the instructions is *This questionnaire asks how you experience the restrictiveness of your care*. For the word *care* there is no corresponding German translation that fits well into the context of a forensic hospital. The usual translation *Pflege* is more commonly used to describe the caring activities for ill people in general hospitals (usually delivered by nurses) or for the elderly. More suiting translations would include *Behandlung* (treatment) or *Unterbringung* (detention). We decided to ask the patients in the pretest which term they would find more appropriate and why.

The second sentence (*We want to know how you feel about each of the statements included below*) referred to the 15 items in which the patients are asked to rate different statements regarding their detention according to their agreement or disagreement. The word *feel* is difficult to translate into German since the literal translation would ask more for an emotional evaluation (e.g., good or bad) and is uncommon in contexts of agreement in German. A better translation would be *halten von* or *denken über* (both translated with *think* in English). During the review discussion the option *halten von* was preferred, since the other option *denken über* was thought to emphasize the cognitive rating over the emotional rating too much. Nonetheless, the translators afterwards mentioned a negative connotation of *halten von* that might influence the patient’s ratings. Therefore, we chose *denken über* for the pretest version.

The last instruction sentence also evoked some discussion and lead to divergent suggestions by the review and the professional translators. The sentence *Please think about how you have felt over the last week when completing this.* Was translated in the review discussion as *Bitte denken Sie beim Ausfüllen daran, wie Sie sich im Verlauf der letzten Woche gefühlt haben*. This sentence contained several difficulties. First, the grammatical structure of the English sentence cannot be copied one by one into German. Second, the word *feel* cannot be translated literally here, since the German question about how somebody feels implies an evaluation (e.g., good, bad) or an emotion (e.g., happy, sad) as an answer. Since the following questionnaire items ask for the amount of agreement to a statement, the word *feel* or its literal translation would not fit into this context and might lead to confusion. Therefore, the word feel was not translated literally as *fühlen* but adapted as *erleben* (experience).

There was also some discussion regarding the translation of the scale points in the third instruction sentence. The scale points were strongly disagree, disagree, not sure, agree und strongly agree. A common German translation is the literal translation *Stimme zu* (agree) and *Stimme voll und ganz zu* (totally agree) and analogue *Stimme nicht zu* and *Stimme überhaupt nicht zu*. One of the professional translators mentioned that this expression indicates a grading of something and that agreement has no grading –you either agree or not. Therefore, she suggested an option that expresses tendencies – *Stimme eher zu* (rather agree) and *Stimme voll zu* (completely agree). We found this explanation reasonable and decided to follow these suggestions.

Further discussion was elicited by the actual questionnaire items. In item 2 (*I can express my feelings here enough*) the review translators discussed the term *enough* and if it might be understood by all patients as intended. That means the patients would be able to express themselves to an extent that they are satisfied (or relieved). The review translators with clinical expertise thought that most of the patients might interpret this item in the intended way. Nonetheless, we decided to evaluate patients’ interpretation of this in the pretest.

Item 3 (*The hospital helps me practice hobbies I like*) led to diverging translations amongst the review translators and objections with the professional translators afterwards. The review translators agreed on the translation *Die Klinik hilft mir dabei, meinen Hobbys nachzugehen* and introduced the term *my hobbies* instead of *hobbies* alone. They intended to remove the term *I like* because they felt like a hobby would be something a person likes *per se*. The professional translators agreed with that. They explained that the term “hobbies” in English would have a broader meaning, compared to German. In English, the word “hobby” includes any kind of leisure time activity. In German, the word “Hobby” also exists but is more linked to leisure time activities that are practiced on a regular basis (e.g., going to the gym twice a week or joining a club’s activities as a registered member). Because of that, they felt that *my hobbies* was not an appropriate translation. The expression *my hobbies* instead of *hobbies* alone might add to a misleading interpretation by patients in a way that they might interpret this item as to which extent they can continue leisure time activities in the clinic that they used to do before detention. This interpretation would lie behind the item’s intention, so we decided to remove the word *my* from the translation and keep a more verbatim translation with *I like (Die Klinik hilft mir dabei, Hobbys nachzugehen, die mir gefallen)*. To test if this translation was still misleading to patients this item should be evaluated in the pretest.

Item 6 (*Staff respect me as an individual*) was translated by in the review translators as *Das Personal respektiert mich als Individuum*. One of the professional translators mentioned that the word *Individuum* is not very common in German everyday language and that forensic patients might have issues with that due to their on average lower level of formal education. Since there is no alternative translation that captures the word’s meaning and is more common in everyday language, we decided to keep the word *Individuum* and test its comprehensibility in the pretest.

A critical word that led to divergent evaluations by review and professional translators was the word *fair* in items 10 (*It is fair I am here right now*) and 14 (*The rules on the ward are fair*). The word *fair* does also exist in German. To stay as close to the original translation as possible and in order to be consistent (both original items used the same word) the review translators kept this word in both translations. The professional translators differed from that and suggested an alternative. They noted that the word fair had a closer interpretation in German compared to English and would be more commonly used in play and sports contexts or when talking about a person’s behavior. Therefore, one of them suggested *gerecht* as more suitable, which can be translated as *just(ified)* or *according to the law* in German. There are also indications in the literature that German participants in a general population survey made a difference between *fair* and *gerecht* (21). Therefore, we decided to further evaluate patients’ interpretations of these items in the pretest.

Issues also arose with item 11 (*I can participate in activities I find meaningful*). The translation of *meaningful* was difficult because review and professional translators were unaware of the original author’s intention. Additionally, the review translator’s version *Ich kann an Aktivitäten teilnehmen, die für mich von Bedeutung sind* was seen as correct in terms of content by the professional translators but they mentioned the expression *die für mich von Bedeutung sind* as slightly uncommon in everyday language in connection with the term *Aktivitäten* (activities). We decided to ask the original author about his intentions regarding the term *meaningful*. He replied that meaningful was meant to express a personal, emotional importance rather than an objective importance. For example, a court appointment might be important objectively but it might not necessarily have the same emotional importance to the patient. Here, e.g., family visits might carry more emotional relevance, even if they are of less objective relevance compared to the court appointment. According to this, we adjusted the initial review translation to *Ich kann an Aktivitäten teilnehmen, die mir wichtig sind* (which was also an alternative suggestion by one of the professional translators). To make sure patients interpret this item in the intended way, we choose to evaluate it in the pretest.

Item 15 (*The restrictions on the ward make sense*) was translated by the review translators as *Die Einschränkungen auf Station sind sinnvoll*. One of the professional translators mentioned that in German make sense is ambiguous. It can address if something is understandable on a cognitive level as well as the evaluation of the reasonableness of the ward’s restrictions. Another consultation with the original questionnaire’s author clarified that the item aims at the latter. The author mentioned an example in which the patients on the ward of a hospital were not allowed to have a knife in their rooms due to reasons of safety but were allowed to have a metal nail file. The patients were able to understand that restriction cognitively but evaluated it negatively since a nail file can also be used as a weapon. As a result, the review translator’s suggestion was kept. To make sure patients interpret this item according to its intention, we decided to evaluate their interpretation in the pretest.

The only item that needed adaptation because of cultural differences was item 4 (*I feel included in my care plan enough [CPA and Ward Rounds]*). The concept of the Care Programme Approach (CPA) is specific to Britain’s health care system and has no equivalent in Germany. Therefore, it was translated in a more general way as *Behandlungsplanung* (treatment planning).

One last adaptation decision was made after the translational process was finished. The reviewer decided, after supervision with the above-mentioned research team member, to remove the demographic items from the questionnaire. There were several reasons for this. First, since the FRQ-G is intended to be used in research primarily, the demographic, legal and clinical variables that have to be collected, vary according to the individual research project’s questions and, therefore, are likely to be changed by other researchers, anyway. Furthermore, in a conversation with the original author it turned out that the demographic variables in his studies were taken from the patients records and not answered by the patients themselves. This was done for reasons of data quality and consistency because it was assumed that some of these questions might be difficult to answer for patients (e.g., for how long they have already been detained and for how long they had been detained previously). The final translation used for the cognitive pretest can be seen in Table 1.

**Table 1:** Original FRQ and German translation after review discussion and adjudication.

English original	German translation
Instructions for use:	Hinweise zum Ausfüllen:
This questionnaire asks how you experience the restrictiveness of your care	In diesem Fragebogen geht es darum, wie Sie die Einschränkungen im Rahmen Ihrer Behandlung erleben
We want to know how you feel about each of the statements included below	Wir möchten erfahren, wie Sie über die untenstehenden Aussagen denken
Please read the following statements and mark ‘Strongly Disagree’, ‘Disagree’, ‘Not Sure, ‘Agree’ or ‘Strongly Agree’	Bitte lesen Sie die folgenden Aussagen und markieren Sie “Stimme überhaupt nicht zu,” “Stimme eher nicht zu“, „Unentschieden“, “Stimme eher zu” und “Stimme voll und ganz zu.”
Please think about how you have felt over the last week when completing this	Bitte denken Sie beim Ausfüllen daran, wie Sie sich im Verlauf der letzten Woche gefühlt haben
If you have any questions, please ask a member of staff	Wenn Sie Fragen haben, wenden Sie sich bitte an das Personal
Information about you:	removed
(To be filled by yourself or member of staff)	removed
Date:	removed
Study ID:	removed
Age:	removed
Ethnicity:	removed
Gender:	removed
Diagnosis:	removed
Mental Health Act Section(s):	removed
How long have you been in this Secure Hospital:	removed
How long have you been in other Secure Hospitals	removed
Index Offence(s) (if applicable):	removed
1. I am treated like a human being here	1. Ich werde hier wie ein Mensch behandelt
2. I can express my feelings here enough	2. Ich habe hier genügend Möglichkeiten, meine Gefühle auszudrücken
3. The hospital helps me practice hobbies I like	3. Die Klinik hilft mir dabei, Hobbys nachzugehen, die mir gefallen
4. I feel included in my care plan enough (CPA and Ward Rounds)	4. Ich fühle mich genügend in meine Behandlung einbezogen (Behandlungsplanung und Visiten)
5. I am given enough information about my care	5. Ich werde genügend über meine Behandlung informiert
6. Staff respect me as an individual	6. Das Personal respektiert mich als Individuum
7. I am given enough responsibility on the ward	7. Mir wird genügend Verantwortung auf Station gegeben
8. I am trusted by staff enough	8. Das Personal vertraut mir genügend
9. I can choose what I want to do each day	9. Ich kann jeden Tag entscheiden, was ich tun möchte
10. It is fair I am here right now	10. Es ist gerecht, dass ich zurzeit hier bin
11. I can participate in activities I find meaningful	11. Ich kann an Aktivitäten teilnehmen, die mir wichtig sind
12. My rights are respected properly here	12. Meine Rechte werden hier angemessen respektiert
13. I am forced to do things I do not want to do	13. Ich werde gezwungen Dinge zu tun, die ich nicht tun will
14. The rules on the ward are fair	14. Die Regeln auf Station sind gerecht
15. The restrictions on the ward make sense	15. Die Einschränkungen auf Station sind sinnvoll

### 3.2. Cognitive pretest

The analysis of the pretest led to several adaptations of the questionnaire. The complete questionnaire after the first and second pretest round is presented in Table 2. For reasons of space only items that led to questionnaire adaptations are discussed. The full results can be provided by the authors upon request.

**Table 2:** Original English Version of the FRQ, review translation and adaptations after the first and second round of pretest.

English Original	German Translation	Adaptations after Pretest, Round 1	Adaptations after Pretest, Round 2
Instructions for use:	Hinweise zum Ausfüllen:	Hinweise zum Ausfüllen:	Hinweise zum Ausfüllen:
This questionnaire asks how you experience the restrictiveness of your *care*.	In diesem Fragebogen geht es darum, wie Sie die Einschränkungen im Rahmen Ihrer *Behandlung* erleben.	In diesem Fragebogen geht es darum, wie Sie die Einschränkungen im Rahmen Ihrer *Unterbringung* erleben.	In diesem Fragebogen geht es darum, wie Sie die Einschränkungen im Rahmen Ihrer *Unterbringung* erleben.
We want to know how you feel about each of the statements included below.	Wir möchten erfahren, wie Sie über die untenstehenden Aussagen denken.	Wir möchten erfahren, wie Sie über die untenstehenden Aussagen denken	Wir möchten erfahren, wie Sie über die untenstehenden Aussagen denken
Please read the following statements and mark ‘Strongly Disagree’, ‘Disagree’, ‘Not Sure, ‘Agree’ or ‘Strongly Agree’.	Bitte lesen Sie die folgenden Aussagen und markieren Sie “Stimme überhaupt nicht zu,” “Stimme eher nicht zu“, „*Unentschieden*“, “Stimme eher zu” und “Stimme voll und ganz zu.”	Bitte lesen Sie die folgenden Aussagen und markieren Sie “Stimme überhaupt nicht zu,” “Stimme eher nicht zu“, „*Teils teils*“, “Stimme eher zu” oder “Stimme voll und ganz zu.”	Bitte lesen Sie die folgenden Aussagen und markieren Sie “Stimme überhaupt nicht zu,” “Stimme eher nicht zu“, „*Teils teils*“, “Stimme eher zu” oder “Stimme voll und ganz zu.”
Please think about how you have felt over the last week when completing this.	Bitte denken Sie beim Ausfüllen daran, wie sie sich im Verlauf der letzten Woche gefühlt haben.	Bitte denken Sie beim Ausfüllen daran, wie Sie sich im Verlauf der letzten Woche gefühlt haben.	Bitte denken Sie beim Ausfüllen daran, wie Sie sich im Verlauf der letzten Woche gefühlt haben.
If you have any questions, please ask a member of staff.	Wenn Sie Fragen haben, wenden Sie sich bitte an das Personal.	Wenn Sie Fragen haben, wenden Sie sich bitte an das Personal.	Wenn Sie Fragen haben, wenden Sie sich bitte an das Personal.
1. I am treated like a human being here	1. Ich werde hier wie ein Mensch behandelt	1. Ich werde hier wie ein Mensch behandelt	1. Ich werde hier wie ein Mensch behandelt
2. I can express my feelings here enough	2. Ich habe hier genügend Möglichkeiten, meine Gefühle auszudrücken	2. Ich habe hier genügend Möglichkeiten, meine Gefühle auszudrücken (B), *wenn ich es möchte* (A)	2. Ich habe hier genügend Möglichkeiten, meine Gefühle auszudrücken, *wenn ich es möchte*
3. The hospital helps me practice *hobbies* I like	3. Die Klinik hilft mir dabei, *Hobbys* nachzugehen, die mir gefallen	3. Die Klinik hilft mir dabei, *Freizeitaktivitäten* (A) / *Hobbys* (B) nachzugehen, die mir gefallen	3. Die Klinik hilft mir dabei, *Freizeitaktivitäten* nachzugehen, die mir gefallen
4. I feel included in my care plan enough (CPA and Ward Rounds)	4. Ich fühle mich genügend in meine Behandlung einbezogen (Behandlungsplanung und Visiten)	4. Ich fühle mich genügend in meine Behandlung einbezogen (Behandlungsplanung und Visiten)	4. Ich fühle mich genügend in meine Behandlung einbezogen (Behandlungsplanung und Visiten)
5. I am given enough information about my care	5. Ich werde genügend über meine Behandlung informiert	5. Ich werde genügend über meine Behandlung informiert	5. Ich werde genügend über meine Behandlung informiert
6. Staff respect me as an *individual*	6. Das Personal respektiert mich als *Individuum*	6. Das Personal respektiert *mich mit all meinen Eigenschaften und Besonderheiten*	6. Das Personal respektiert mich *als Persönlichkeit mit all meinen Eigenschaften*
7. I am given enough responsibility on the ward	7. Mir wird genügend Verantwortung auf Station gegeben	7. Mir wird genügend Verantwortung auf Station gegeben	7. Mir wird genügend Verantwortung auf Station gegeben
8. I am trusted by staff enough	8. Das Personal vertraut mir genügend	8. Das Personal vertraut mir genügend	8. Das Personal vertraut mir genügend
9. I can choose what I want to do each day	9. Ich kann jeden Tag entscheiden, was ich tun möchte	9. Ich kann jeden Tag entscheiden, was ich tun möchte	9. Ich kann jeden Tag entscheiden, was ich tun möchte
10. It is *fair* I am here right now	10. Es ist *gerecht*, dass ich zurzeit hier bin	10. Es ist *gerecht* (A)/*fair* (B), dass ich zurzeit hier bin.	10. Es ist *gerecht*, dass ich zurzeit hier bin
11. I can participate in activities I find meaningful	11. Ich kann an Aktivitäten teilnehmen, die mir wichtig sind	11. Ich kann an Aktivitäten teilnehmen, die mir wichtig sind	11. Ich kann an Aktivitäten teilnehmen, die mir wichtig sind
12. My rights are respected properly here	12. Meine Rechte werden hier angemessen respektiert	12. Meine Rechte werden hier angemessen respektiert	12. Meine Rechte werden hier angemessen respektiert
13. I am forced to do things I do not want to do	13. Ich werde gezwungen Dinge zu tun, die ich nicht tun will	13. Ich werde gezwungen Dinge zu tun, die ich nicht tun will	13. Ich werde gezwungen Dinge zu tun, die ich nicht tun will
14. The rules on the ward are *fair*	14. Die Regeln auf Station sind *gerecht*	14. Die Regeln auf Station sind *gerecht* (A) / *fair* (B)	14. Die Regeln auf Station sind *fair*
15. The restrictions on the ward make sense	15. Die Einschränkungen auf Station sind sinnvoll	15. Die Einschränkungen auf Station sind sinnvoll	15. Die Einschränkungen auf Station sind sinnvoll

#### 3.2.1. Differences between *Behandlung* and *Unterbringung*

In the pretest patients were given the questionnaire sentence on a showcard either with the word *Unterbringung* or *Behandlung*. After reading this sentence, another showcard was presented with both, the initial and the alternative option. Patients were asked if they see a difference in the meaning of the two sentences. Four of nine patients stated that they would not see a difference in the meaning (two of them had the initial sentence with *Behandlung*, two the alternative with *Unterbringung*). Five patients stated that for them there is a difference. Three of them had the initial option with *Unterbringung* (detention), two the alternative with *Behandlung* (treatment). Patients who made a difference were asked for more explanations. Three of the patients stated that they see *Unterbringung* as a broader term, that would include their treatment (in form of therapeutic activities, e.g., sessions with their psychologist, occupational therapy) and their spare time (e.g., visits by family members or having leave). Furthermore, for two patients the word *Unterbringung* had a more legal connotation and was associated with the court or the court order. One patient interpreted the word *Behandlung* as the way patients were treated generally (i.e., beyond the therapeutic interventions). This kind of interpretation makes sense in German but goes beyond the original intention of the question. Since the broader interpretation of *Unterbringung* and the potential for interpreting *Behandlung* in a non-intended way, we decided to use the term *Unterbringung* in the questionnaire. This question was not incluced in the second round of the pretest.

#### 3.2.2. Item 2 (I can express my feelings here enough)

In Item 2 a literal translation was possible. In the reviewer discussion it was mentioned that the word *enough* might lead to confusion amongst patients. Therefore, a category selection probe and, if necessary, a specific probe was used to explore patients’ interpretation of this statement. It was anticipated that, if patients understand the item according to the author’s intention, a low rating (strongly disagree, disagree a little) would correspond to a low number of opportunities for expression of feelings, and a high rating (agree a little, strongly agree) would correspond to sufficient opportunities. From nine patients only one gave a low rating, giving as a reason the high fluctuation of staff at his ward at the time, including therapists and nurses, so he had to deal with most of his emotions by himself. Compared to this, four patients stated to agree (one patient strongly, three a little). They said that there were opportunities to express their feelings anytime and with several people (fellow patients or staff) or that they could get an additional appointment with their therapist very soon, if needed. Interestingly, the patients who stated to not be sure about this (rating of 3) stated that they were not sure about their feelings oftentimes and would generally prefer not to talk about them. On further questioning all of them stated that they felt that there was (at least) one person to turn to in case they needed someone to talk to, even if this was seldom the case. We interpreted the answers of these three patients in a way that some patients were not used to talk about their feelings and did not use this option often. Therefore, they might have used the middle category as an alternative for *I do not know what to answer*. Since this behavior, if used by other patients, too, could lead to an overestimation of the middle category, we decided to adapt Item 2 by the phrase *wenn ich es möchte* (if I like to).

The original translation was tested against the adapted version in pretest round two. In two versions, half of the patients received the original translation and the other half the adapted translation. We used the same probes as in round one to explore patients’ interpretation of the item. Of the eight participants of the second pretest-round, seven gave a rating that was in line with the expected interpretation. Three patients rated this item on the middle category. All of them had the adapted version. Two patients stated that the expression of their feelings would not always be possible because a person of trust was sometimes not available (e.g., due to work schedule, illness or holiday). The remaining patient had the adapted version of the pretest. He stated that he would have difficulties in expressing especially negative emotions to others and he never did that in detention at the time of the interview. Therefore, answering the question would be speculative, even when he stated there would be some people who he could imagine to turn to. This patient used the middle category due to the absence of a more suitable answering option.

In the end, we decided to keep the adjusted item with the added phrase *wenn ich es möchte* (if I like to). We think that this phrase can guide the answering behavior of patients who are not overly used to express their feelings but did that in the past. Nonetheless, this item could be difficult for patients who never express their feelings at all. These patients might use the middle answer category, as the patient in round two, or leave the question unanswered. The latter could be detected in an increased rate of missing answers in the pilot test.

#### 3.2.3. Item 3 (The hospital helps me practice hobbies I like)

According to the professional translator’s objection, we wanted to evaluate how patients interpret the term *Hobbys*. If patients’ interpretation was according to the original item’s intention, patients should think predominantly about leisure time activities they can do during their detention in the clinic. We evaluated this question using category selection and specific probes. In the first pretest round it turned out that patients indeed mixed-up activities they were offered in the clinic (e.g., table tennis, the clinic’s gym) and activities they used to do before their detention but were not during their stay in the clinic (e.g., football, playing darts). Therefore, we decided to adapt the term *hobbys* by the term *Freizeitaktivitäten* (leisure time activity) that better reflects the emphasis on clinic-based activities. The new wording was evaluated in the second round of the pretest.

Again, we tested the original translation with *Hobbys* against the adapted version with *Freizeitaktivitäten* in pretest round two with half of the patients receiving the original translation and the other half the adapted translation. The same probes as in round one were used. From eight patients six focused on activities that are possible within the clinic. The remaining two patients additionally mentioned some activities they used to do before their detention. One of them was able to continue practicing this hobby (making music) in the clinic. The different wordings seemed to have no influence on the answering behavior, patients’ reasoning was in line with the intention of the item.

The issues found in pretest round one could not be found in round two. This could be a reflection of the higher intellectual abilities of the second sample (see WAIS-IV scores in Supplementary Appendix A). Since both wordings were well understood in round two and the wording *Hobbys* seemed an issue in round one, we decided to keep the alternative *Freizeitaktivitäten* (leisure time activities) for the pilot study.

#### 3.2.4. Item 4 (I feel included in my care plan enough (CPA and Ward rounds))

The aim of this pretest was, similar to Item 2, to explore how patients interpret the term *enough*. According to the test of item 2, we expected low ratings of agreement to correspond with the report of limited inclusion (e.g., in form of communication, shared decision making or similar) in the probes and vice versa. Of nine patients, two gave low ratings of agreement, one patient used the middle category and the remaining six patients gave high ratings of (strong or a little) agreement. The following probes revealed that especially patients who agreed on this item predominantly mentioned the good information about their care. Since this is actually the content of Item 5 (*I am given enough information about my care*) we saw this evaluation as biased. This result might not have occurred if patients had known item 5 before, so we decided to ask the same probes of Item 4 again in the second pretest round but switch the order of Item 4 and Item 5. By doing so, we hoped that being aware of the content of Item 5 before Item 4 might lead patients to the intended interpretation of inclusion into their care so that the initially intended test of *enough* would become possible.

In round two, two patients disagreed to feeling included in their care plan whereas four agreed and two strongly agreed. Patients who disagreed stated that they did not have the feeling to be heard or got no or late information about their results of medical examinations. Furthermore, organizational issues were mentioned, e.g., not having a care plan due to frequent staff changes. In contrast, patients who agreed with that item reported regular meetings with staff in which they could talk about their treatment. Patients who strongly agreed also mentioned their opinion was heard when it came to treatment decisions. On further questioning, all of these six patients indicated their wishes regarding their treatment were respected.

The second pretest round indicated that patients interpret the item as intended. Therefore, no adaptations to item 4 appeared necessary. Nonetheless, we decided to switch items 4 and 5 in the original survey to avoid the positioning effects that were observed in the pretest.

#### 3.2.5. Item 6 (Staff respect me as an individual)

We used comprehension probes and category selection probes to explore how patients might understand the word *Individuum*. The pretest confirmed the professional translator’s presumption. Of nine patients, five spontaneously asked about the word’s meaning after hearing the Item. From the remaining three patients, only two gave an explanation of *Individuum* that was on target. One patient interpreted *Individuum* incorrectly. In conclusion, six of nine patients had issues understanding the word *Individuum*. Therefore, we adapted the item and evaluated the adaptation in the second round of the pretest.

In the second pretest round, we replaced the word *Individuum* (individual) by an explanation that followed the explanation of the Duden (German dictionary). The adjusted item was *Das Personal respektiert mich mit all meinen Eigenschaften und Besonderheiten* (Staff respect me with all my qualities and characteristics). None of the patients indicated issues in understanding there. Four of them agreed partially, three agreed and one patient agreed strongly. Again, we used a category selection probe to see how they came to their conclusion. Patients’ answers indicated that they interpreted the item as intended. Patients who agreed (strongly) stated, e.g., that they feel respected by staff the way they are and had a good relationship with them. Patients who agreed partly stated that this would apply at least for some members of staff. One patient additionally mentioned that the word *Besonderheiten* (characteristics) had a negative connotation to him. He explained that this could be interpreted like being special or “nuts.” Instead of that, he suggested *Das Personal respektiert mich als Persönlichkeit* (Staff respect me as a personality). We discussed that point during the analysis and agreed with the patient’s view that an unintended negative connotation can be seen in this translation. Therefore, we adjusted the item again to *Das Personal respektiert mich als Persönlichkeit mit all meinen Eigenschaften* (about: Staff respect me as a personality with all my qualities). This version is based on the patient’s suggestion and the Duden definition.

#### 3.2.6. Items 10 and 14 (It is fair I am here right now and the rules on the ward are fair)

The critical word in these items was *fair*, which can be translated into German literally as *fair* or – according to the context – as *gerecht* (just). Item 10 refers more to patients’ evaluation if their detention was justified, whereas Item 14 aims more at patients’ perception of the appropriateness of the wards rules and if they feel these rules are applied to all patients equally.

To explore if patients answering the FRQ also make a difference between the two terms *fair* and *gerecht*, we presented items 10 and 14 in both versions. Half of the pretest patients in round one were initially presented a version with *fair*, the remaining four patients with *gerecht*. After their initial rating, participants were presented the alternative and were asked if they had answered this question differently and if they saw a difference between the words *fair* and *gerecht*. Of nine patients in round one, eight replied that they would not answer the alternative question differently from the first one in Item 10. Nonetheless, three stated they see a difference between the words. These patients associated *gerecht* with reasonable consequences for their offences or justified by law. One of these patients noted that for them *gerecht* is more a term association with reason or cognition, *fair* more with emotional or sympathetic decision making. Two patients interpreted *gerecht* in a way that their detention is justified and that they can accept it. Defining the word *fair* was found to be difficult by the patients and only two were able to find a description. One described it as *fair* to be sent to a forensic hospital by court. The other one found *fair* to have a more positive, benevolent connotation than *gerecht*. In Item 14 only eight patients could answer this item (see below for explanation). From the eight patients seven replied they would not answer the alternative item differently from the initial wording. One patient stated he would answer both items differently. Again, three patients indicated they see a difference between *fair* and *gerecht*, although for one patient the difference was very small. Their explanations diverged. One patient described *gerecht* as a more objective term, whereas *fair* felt more subjective for him. The other two patients associated *gerecht* with consequences for violating rules and the absence of harassing behavior of staff. One of them additionally mentioned that fair would mean to treat everybody the same way.

In general, patients in round one described the terms *fair* and *gerecht* in a quite diverse way. Some of their descriptions were in line with our expectations, e.g., the association between *gerecht* and (objective, justified) court ruling where people receive punishment when breaking the law or *fair* to be more related to interpersonal fairness and treating everybody the same way. On the other way, patients also mentioned interpretations outside of our expectations. Because of the diverse interpretations and the difficulties to describe the term *fair*, we decided to test the item again in the second round to see if other patients agree with that interpretation.

The same procedure as in round one was applied in round two. Half of the patients were presented the version with *fair* first and then asked for the alternative with *gerecht* and the other half had the switched word order. Furthermore, we now asked all patients for their interpretation of *fair* and *gerecht*, and left out the question if they would see a difference between these terms.

For item 10 (*It is fair that I am here right now*) we found a larger variance in the answers in round two than in round one. Five patients interpreted the word *gerecht* in the legal context, e.g., they stated they accept the sentence to be in a forensic psychiatric hospital as justified and deserved. Another two patients mentioned a more distant legal context. One of them discussed the sentence in relation to the two people who were accomplices in his offence. Unlike his accomplices, who were sentenced to prison, the patient received a prison sentence and in addition was sent to a forensic psychiatric hospital, which he considered as unfair *(ungerecht)*. The other patient discussed the length of his sentence in relation to his victim’s injuries. He felt the sentence length was disproportionate and therefore – to some extent – unfair (*ungerecht*). The remaining two patients discussed *gerecht* in a therapeutic interpersonal context. They were in forensic treatment repeatedly and considered that potentially unfair compared to other addicted offenders who offended for the first time. These people might also need treatment in a forensic psychiatric setting but could not be offered this because of the limited resources were are already taken up by them (the participating patients). The same explanation was given by three patients about their interpretation of the German word *fair*. They considered it *unfair* that they, as being repeatedly in forensic treatment, would take away a therapeutic option for first time offenders with a substance use disorder. The German *fair* was also mentioned by four patients in the context that they felt it was *fair* that their addiction was considered by the court and that they were given the opportunity for (forensic) treatment instead of a prison sentence only. One patient interpreted the German *fair* as justified, which implies a legal context.

Taken together both pretest rounds, for item 10 the majority of patients interpreted *gerecht* in a legal context as being justified or having a legal basis. *Fair* was more seen in an interpersonal context or in a way that individual circumstances were taken into consideration by the court. Other explanations also occurred that were not in line with our expectations.

Since the original item aims more for the patient’s view on (legal) justice, which was mentioned by the professional translators as well as a reasonable number of patients, we decided for the alternative *gerecht* in item 10 (*Es ist gerecht, dass ich zur Zeit hier bin*).

With item 14 (*The rules on the ward are fair*) patients’ answers indicate that *gerecht* is more interpreted in the context of formal rules and that their violation has consequences whereas *fair* was described in several ways, e.g., as interpersonal fairness, the option to negotiate ward rules with staff but also as having consequences when violating ward rules.

Since the original item’s intention aimed more for perception of interpersonal fairness and humane interaction than sticking to formal rules, the alternative *fair* appeared to be more appropriate for us. Unlike our initial intention for consistency, we decided to accept a different wording than in item 10 and choose the alternative *fair* for the FRQ (*Die Regeln auf Station sind fair*).

Furthermore, Item 14 revealed an unintended point that might be an issue and was not recognized by the translators before. This was the inappropriateness of some of the items for patients who are very advanced in their therapeutic process and might be allowed to leave the hospital for longer periods or reside outside the main hospital. Since these patients are visited by clinical staff at the facility they currently live in, they might not have contact to their former wards on a regular basis anymore. For these patients, items asking about their experiences during the last week on the ward does not make sense. These circumstances applied for one patient in pretest round one, who could not answer this item. We decided to take this into consideration in pretest round two and not invite patients in external living facilities to take part.

#### 3.2.7. 5-Point Likert scale, middle scale point (not sure)

During the first pretest round it turned out that some patients found the middle scale point to be inappropriate. For example, in Items 1 (*I am treated like a human being here*), 6 (*Staff respect me as an individual*), 8 (*I am trusted by staff enough*), and 12 (*My rights are respected properly here*) some patients indicated that there were differences in how they were treated by different members of staff or that some statements apply to their situation only sometimes. If the statement would apply for about half of the staff or about half the time, they tended to use the middle category. In that case, the German description of the middle scale point as *unentschieden* (undecided) did not match these thoughts. As one patient stated, he was not undecided, but had a clear opinion. Another patient mentioned that (regarding the hospital staff) some of them act in one way and some in another way. We saw the patients points as reasonable and, therefore, adjusted the middle scale point from *unentschieden* (undecided) to *teils teils* (partly). The term *teils teils* is a common alternative for the middle category in German questionnaires so we saw this as more appropriate and also as easy to accept by researchers and research participants. No issues occurred during the second round of the pretest so this adaptation was kept.

## 4. Discussion

This article describes the TRAPD approach developed by Harkness (13, 22) as a translation approach and its application for the translation of the Forensic Restrictiveness Questionnaire [FRQ; (14)] from English into German. In this approach, TRAPD is an acronym for the five steps of the process, namely translation, review, adjudication, pretest and documentation.

According to this approach, several translators with expertise in different fields (e.g., clinical, linguistic, cultural) translated the questionnaire first by themselves and then discussed the individual versions in a shared review discussion. The discussion was moderated by a person who was familiar with the overall project. The review moderator along with an adjudicator, who was also familiar with the project but not involved in the translation process so far, made the final decision about the translation. This draft was applied in a pretest with 17 forensic mental health patients in two rounds (with 9 and 8 patients each). The review discussion and the pretest rounds led to adaptations of the questionnaire, e.g., due to cultural differences, unintended implicit assumptions or ambiguous or difficult word choices. The result will be tested for its psychometric properties in a larger pilot study.

During the translational process the necessity and usefulness of different backgrounds of translators became obvious. The clinical expertise of two of the review translators supported appropriate cultural and contextual adaptations that could not be covered by the other review translators or the professional translators (e.g., finding an appropriate equivalent for CPA in item 4, which is a special component of Britain’s health care system that has no equivalent in Germany). The lay translators’ translations tended to be shorter and more oriented towards everyday language that might be easier to understand and, therefore, more acceptable for patients. Finally, the professional translators contributed their expertise regarding linguistic topics like grammar, sentence structure and connotation. They were able to identify ambiguous wordings in the original or translated questionnaire (e.g., *makes sense* in item 15) as well as unintended connotations of words and phrases (e.g., in the translation of *how you feel about* in introductory sentence 2) and recognized potential issues with translated items due to unintended implicit assumptions (like the translation of *hobbies* in item 3). Furthermore, they had a better understanding of words that sound the same but have a broader or closer translation in one language (e.g., *hobby* in English versus *Hobby* in German, where Hobby has a closer meaning or *fair*, which has also a broader range of interpretations in English than it has in German). Combining all of this expertise enabled a translation ready for pretesting with the intended user population of forensic mental health patients.

The pretest with forensic mental health patients was done to check the professional translation for its comprehensibility. It revealed also further adaptation necessities. In some cases, patients’ answers confirmed assumptions that review or professional translators had before, e.g., that a literal translation of the English *individual* to *Individuum* in German (item 6) was not understood by a reasonable number of patients and, therefore, had to be altered. In other cases, patients pointed out shortcomings none of the translators had identified before, e.g., regarding an alternative translation of the Likert scale’s midpoint that made more sense in their context or that some items are inappropriate for some patients, e.g., items 7, 14, and 15 which are statements about things on the ward and, therefore, are difficult to answer for patients already on a high level of leave and living in external facilities.

We conclude that the participation of translators with various backgrounds and a pretest with the intended user group (in this case, with forensic mental health patients) both added valuable input to the translation of the FRQ. Nonetheless, some limitations should be discussed. The number of translators who participated in that translation process was quite high. Also, the original TRAPD approach does not require an evaluation of the translation after the review discussion by further translators. This was done as an additional step of quality assurance. Since the professional translators added reasonable thoughts and ideas, it appears useful to include them in the team of initial translators so they can participate in the review discussion. Other limitations include the patient sample of the pretest. Despite an initial sampling plan, some subgroups were difficult to recruit for the patient pretest, especially women (one participant), but also patients with a migration background (two participants) who were underrepresented in the sample. Also, patients with schizophrenia were underrepresented (two participants). Since schizophrenia has an impact on peoples’ thinking (23), we cannot rule out that some patients with schizophrenia might have issues in answering the FRQ. The fact that the FRQ is intended for respondents who are able to give informed consent might work against this. Therefore, the application of the FRQ will require careful participant selection with support of clinicians. The limited representation of these groups of patients is seen as a result of several factors. First, the treatment emphasis of the hospital the patients were recruited was on offender patients with substance use disorders. Therefore, the number of patients with schizophrenia was quite low. Second, the general proportion of females in forensic mental health institutions is quite low [about 5–8%, (Statistisches (15, 24))]. Furthermore, the clinic is located in a geographical an area with a quite low proportion of people with migration background (and hence, potential patients), compared to the rest of Germany.

Another limitation relates to the empirical background of the TRAPD approach. The TRAPD approach or comparable team approaches appear to be seldom used in psychological questionnaire translation but more often used in international survey research [e.g., (25)]. We found almost no research on TRAPD or other team-based approaches being compared to more established translation approaches. This might be the reason why many translation guidelines for surveys and questionnaires do not make specific suggestions [e.g., (7)]. There is some evidence that team-based approaches, such as the TRAPD, deliver the same or, in some points even superior results (26, 27). Since the aim of this paper was the depiction of the TRAPD approach rather than a methodological comparison, we cannot say if a translation would have been superior if we used another approach. More research is needed that compares the several procedures and evaluate which one delivers reasonable translations along with an appropriate relation of effort and results.

## Data availability statement

The raw data supporting the conclusions of this article will be made available by the authors, without undue reservation.

## Ethics statement

The studies involving human participants were reviewed and approved by Ethics Committee at the Rostock University Medical Center. The patients/participants provided their written informed consent to participate in this study.

## Author contributions

PW and BV contributed to conception and design of the study. PW organized and conducted the data collection and analysis and wrote the first draft of the manuscript. BV supervised the data collection and analysis process. All authors contributed to the article and approved the submitted version.

## Conflict of interest

The authors declare that the research was conducted in the absence of any commercial or financial relationships that could be construed as a potential conflict of interest.

## Publisher’s note

All claims expressed in this article are solely those of the authors and do not necessarily represent those of their affiliated organizations, or those of the publisher, the editors and the reviewers. Any product that may be evaluated in this article, or claim that may be made by its manufacturer, is not guaranteed or endorsed by the publisher.
