# Supplementary material for: The TRAPD approach as a method for questionnaire translation
Source: Front Psychiatry. 2023 Jun 15;14:1199989. doi: 10.3389/fpsyt.2023.1199989 (PMC10309006; doi:10.3389/fpsyt.2023.1199989)
Supplement: Supplementary file 1 [file Data_Sheet_1.PDF]

Appendix A

Characteristics of patients who participated in the pretest

Participant Number	Sex	Age	Legal section of detention	Diagnosis	WAIS-IV	Migration Background	Version A (Behandlung, gerecht) / B (Unterbringung, fair)
**Pretest Round 1**							
01	M	56	§ 64 StGB	Substance Use Disorder<br>Personality Disorder<br>Other	74	none	B
02	M	46	§ 64 StGB	Substance Use Disorder<br>Personality Disorder	116	none	A
03	M	23	§ 63 StGB	Substance Use Disorder<br>Personality Disorder<br>Other	66	none	B
04	M	25	§ 64 StGB	Substance Use Disorder	67	none	A
05	M	29	§ 63 StGB	Intellectual Disability	67	none	A
06	M	20	§ 64 StGB	Substance Use Disorder<br>Other	69	none	B
07	M	38	§ 64 StGB	Substance Use Disorder<br>Personality Disorder<br>Other	68	none	B
08	M	24	§ 64 StGB	Substance Use Disorder<br>Other	73	none	A
09	M	31	§ 63 StGB	Schizophrenia<br>Substance Use Disorder	71	none	B
**Pretest Round 2**							
10	M	62	§63 StGB	Schizophrenia	106	none	B
11	F	23	§63 StGB	Personality Disorder	67	none	A

12	M	29	§ 64 StGB	Substance Use Disorder<br>Personality Disorder	93	none	A
13	M	41	§ 64 StGB	Substance Use Disorder	91	AK – 62 (95%-CI 53-71) [a]<br>AI – 64 (95%-CI 58-70) [a]	B
14	M	49	§ 64 StGB	Substance Use Disorder	114	none	A
15	M	36	§ 64 StGB	Substance Use Disorder	97	none	B
16	M	37	§ 64 StGB	Substance Use Disorder	100	none	A
17	M	30	§ 64 StGB	Substance Use Disorder<br>Personality Disorder	96	AK – 63 (95%-CI 54-72) [a]<br>AI – 68 (95%-CI 62-74) [a]	B

Note. [a] T-Values und 95%-Confidence Intervals in brackets at the Frankfurter Akkulturationsskala (FRAKK; Bongard et al., 2020) describe both patient's orientation to German culture (Aufnahmekultur, AK) as average or above average. That means, both patients describe themselves as attached to German culture and identify themselves with it. Furthermore, both patients describe an assimilation index (Assimilationsindex, AI) on average or above average. That means, the patients describe their identity as completely integrated into German society. People with high AI live like German people and don't distinguish from people of German origin in the way they experience or behave. Therefore, both patients are appropriate participants for the pretest.
